# The Regulatory Roles of Intrinsically Disordered Linker in VRN1-DNA Phase Separation

**DOI:** 10.3390/ijms23094594

**Published:** 2022-04-21

**Authors:** Qiaojing Huang, Yanyan Wang, Zhirong Liu, Luhua Lai

**Affiliations:** 1 Beijing National Laboratory for Molecular Sciences (BNLMS), State Key Laboratory for Structural Chemistry of Unstable and Stable Species, College of Chemistry and Molecular Engineering, Peking University, Beijing 100871, China; chemhqj@pku.edu.cn; 2 Peking-Tsinghua Center for Life Sciences, Peking University, Beijing 100871, China; wangyanyan@pku.edu.cn; 3 Center for Quantitative Biology, Peking University, Beijing 100871, China; 4 Research Unit of Drug Design Method, Chinese Academy of Medical Sciences (2021RU014), Beijing 100871, China

**Keywords:** transcriptional repressor VRN1, intrinsically disordered linker, phase separation, polymer chain model, Monte-Carlo simulations, lattice model, effective local concentration

## Abstract

Biomacromolecules often form condensates to function in cells. VRN1 is a transcriptional repressor that plays a key role in plant vernalization. Containing two DNA-binding domains connected by an intrinsically disordered linker (IDL), VRN1 was shown to undergo liquid-like phase separation with DNA, and the length and charge pattern of IDL play major regulatory roles. However, the underlying mechanism remains elusive. Using a polymer chain model and lattice-based Monte-Carlo simulations, we comprehensively investigated how the IDL regulates VRN1 and DNA phase separation. Using a worm-like chain model, we showed that the IDL controls the binding affinity of VRN1 to DNA, by modulating the effective local concentration of the VRN1 DNA-binding domains. The predicted binding affinities, under different IDL lengths, were in good agreement with previously reported experimental results. Our simulation of the phase diagrams of the VRN1 variants with neutral IDLs and DNA revealed that the ability of phase separation first increased and then decreased, along with the increase in the linker length. The strongest phase separation ability was achieved when the linker length was between 40 and 80 residues long. Adding charged patches to the IDL resulted in robust phase separation that changed little with IDL length variations. Our study provides mechanism insights on how IDL regulates VRN1 and DNA phase separation, and why naturally occurring VRN1-like proteins evolve to contain the charge segregated IDL sequences, which may also shed light on the molecular mechanisms of other IDL-regulated phase separation processes in living cells.

## 1. Introduction

Living cells contain huge amounts of biomacromolecules in a limited space, which often form membraneless organelles to mediate a myriad of functions precisely and efficiently, such as chromatin remodeling [1,2,3,4], gene transcription [5] and biomolecule sequestration [6]. Since the first observation of liquid-liquid phase separation (LLPS) in germline P granules [7], more and more evidence suggests that liquid-like droplets are widely spread and utilized in both eukaryotic and prokaryotic cells [8,9], by virtue of achieving complex multicomponent subcellular translocation and enhanced local concentration, making them a promising approach to ensure homeostasis and development. The dysfunction of LLPS will induce severe diseases [10,11,12], such as neurodegenerative diseases. To integrate these intensive data, several databases [13] have been built to record the LLPS-related biological functions, regulating mechanisms, components and subcellular locations in cells, such as LLPSDB [14], DrLLPS [15], PhaSePro [16] and PhaSepDB [17]. Based on these invaluable resources, machine learning-based algorithms have also been developed to predict LLPS proteins [18,19].

The major driving forces of LLPS are supposed to be the non-specific interactions between biomolecules; however, specific interactions can also provide heterogeneity for these biomolecular condensates [20,21], especially for those that possess weak multivalent interactions [21,22,23], such as intrinsically disordered proteins (IDPs) or intrinsically disordered regions (IDRs), which lack well-defined structures and are frequently observed in the liquid-like droplets [9,24]. IDRs often act as disordered linkers to connect and interact with globular domains in multidomain proteins [25], the major protein architecture in LLPS. The sequence pattern and length of disordered linkers regulate the interactions between domains [26,27,28,29] and modulate macroscopic phase behaviors, such as liquid-like droplets and aggregates [30,31,32]. These features can, in turn, guide the design of the linkers with specific lengths and charge patterns to artificially modulate LLPS [33] and the binding affinity between domains. In general, multidomain proteins can be divided into stickers, which form specific and multivalent interactions that mainly correspond to domains, and spacers that provide non-specific interactions or a volume exclusion effect, corresponding to the neutral disordered linkers [30,34,35]. The sticker-spacer framework [36] has deepened the understanding of LLPS and successfully revealed the underlying mechanism of some complex phase separation phenomenon, using both computational and experimental techniques [37,38,39].

The transcriptional repressor VRN1 is a multidomain DNA-binding protein involved in plant vernalization [40,41,42]. VRN1 contains two B3 domains, connected by an intrinsically disordered linker (IDL), which can bind DNA to undergo LLPS [43]. A previous study showed that the length and charge pattern of the IDL could modulate both the binding affinity of the B3 domain to DNA and the phase separation behavior, which changes from gel-like aggregates to liquid-like droplets and solution, depending on the linker length and existence of charged patches [44]. This IDL charge segregation pattern is also conserved in the transcriptional activation domains of many transcriptional factors, hinting a general property [45]. However, it is still obscure how the linker length and charge segregation modulate these mesoscale behaviors, hindering the cognition and application of these rules. 

To answer these questions, we used a polymer chain model and lattice-based Monte-Carlo simulations to comprehensively investigate how the IDL mediates VRN1 and DNA phase separation. The IDL length and sequence pattern control the effective local concentration of the DNA-binding domains and the binding free energy between VRN1 and DNA, which further affects the macroscopic phase behavior. The simulated phase diagrams under different conditions also qualitatively reproduced the previous experimental results [44]. These findings not only explain the experimental observations, but also deepen our understanding of IDL-regulated phase separation processes in cells.

## 2. Results

### 2.1. The Effective Local Concentration Effect of IDL has Essential Influence on the Binding Affinity between VRN1 and DNA

**Figure 1:**
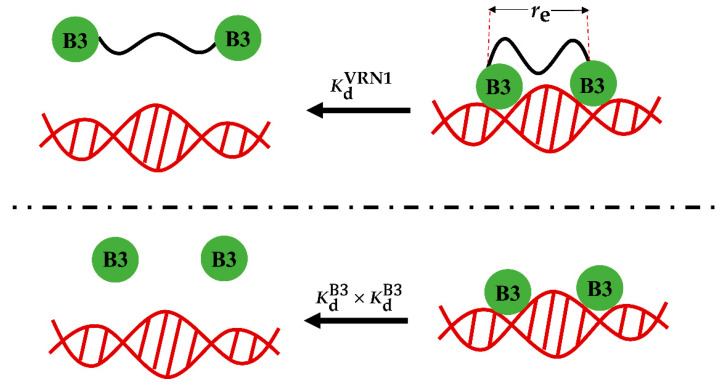
Schematic diagram of *C*_eff_ calculations. Top: two B3 domains in VRN1 bind to DNA cooperatively, the dissociation constant of which can be quantitated by $K_{d}^{\mathrm{VRN}1}$. $r_{e}$ denotes the end-to-end distance of the linker in the binding complex. Bottom: two B3 domains bind to DNA independently, the dissociation constant of which can be quantitated by $K_{d}^{B3}{\times K}_{d}^{B3}$. The linker in VRN1 provides effective local concentration to enhance the binding of VRN1 to DNA, which can be quantitated by the ratio of $\frac{K_{d}^{B3}{\times K}_{d}^{B3}}{K_{d}^{\mathrm{VRN}1}}$.

**Figure 2:**
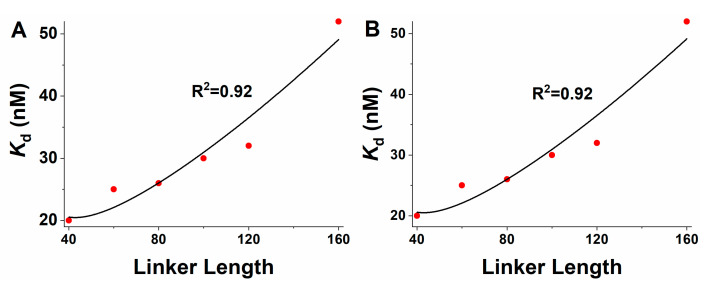
Fitted correlation between the VRN1 linker length and binding affinity to DNA. The linker length is measured by the number of residues. Scatterings are experimental data, as listed in Table 1. Solid lines are the fitting results by (**A**) the WLC model and (**B**) the RMC model with Equations (2) and (4). The resulting fitted parameters are $r_{e}=$ 31.6 Å and $p_{0}=0.007$ in (**A**) and $r_{e}=24.9$ Å and $p_{0}=0.004$ in (**B**).

In domain-linker-domain (DLD) protein architectures, the linkers enhance the local concentration of domains to allosterically promote ligand binding [25,26], which can be described as a worm-like chain (WLC) [29,46,47,48] or a random-coil chain (RMC) [49]. VRN1 has a typical DLD architecture. In order to study the length effect of the IDL, Wang et al. constructed a series of VRN1 variants (PSN) with different lengths of IDL, containing PS repeats, and found that their apparent binding affinity with DNA decreases with the increase in IDL length, as summarized in Table 1 [44]. As these linkers are composed of neutral residues and do not interact with the surroundings, the entropic effect may be dominant and polymer chain models can be applied. The binding of VRN1 to DNA is much stronger than that of a single B3 domain, due to the enhanced local concentration effect, as shown in Figure 1. The apparent VRN1-DNA binding affinity is related to the effective local concentration caused by the linker, which can be calculated using the following equation:

(1) $$C_{\mathrm{eff}}=\frac{K_{d}^{B3}{\times K}_{d}^{B3}}{K_{d}^{\mathrm{VRN}1}}\;$$

where $K_{d}^{B3}$ is the binding affinity of a single B3 domain to DNA (753 nM) and $K_{d}^{\mathrm{VRN}1}$ is the binding affinity between VRN1 variants, with different linker lengths and DNA (Table 1). $C_{\mathrm{eff}}$ is expected to depend mainly on the linker length, as described in Section 4. To test whether the linker-length dependence of the VRN1-DNA affinity can be explained by such a mechanism, the experimental data were fitted with WLC and RMC models. In both models, there were only the following two fitted parameters: the end-to-end distance ($r_{e}$) of the IDL in the VRN1-DNA complex and a proportional factor ($p_{0}$). It was shown that the experimental results can be fitted well by both models (Figure 2), with the Pearson correlation coefficient of 0.92. Therefore, the IDL enhances the VRN1-DNA binding affinity via the effective local concentration $C_{\mathrm{eff}}$. A longer VRN1 IDL leads to a lower $C_{\mathrm{eff}}$; thus, causes weaker binding to DNA. The predicted end-to-end distance is $r_{e}=$ 31.6 Å for WLC, being close to the predicted $r_{e}=24.9$ Å for RMC.

**Figure 3:**
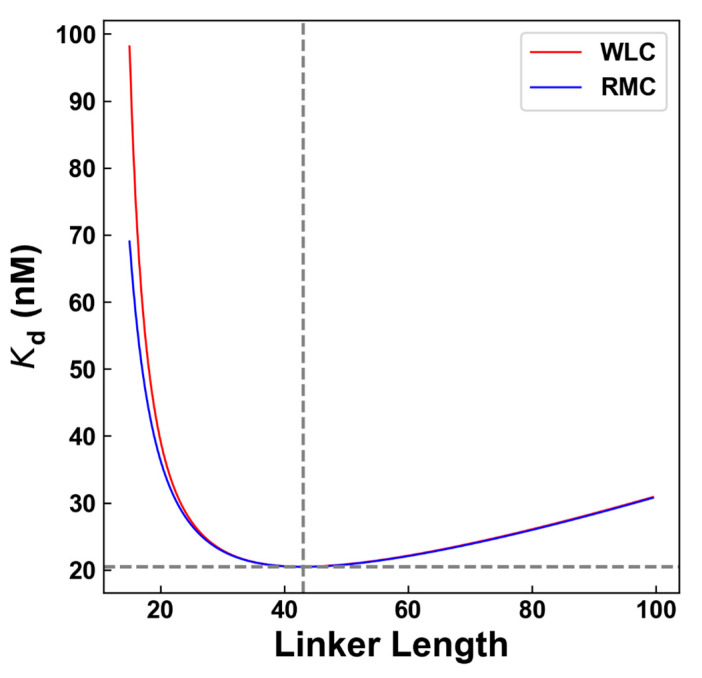
Prediction of the optimal IDL linker length to achieve the strongest binding affinity to DNA, using a WLC model (red line) and a RMC model (blue line). The dotted grey line indicated the optimal linker length (43 residues), under which the predicted $K_{d}$ is the minimum (20.5 nM).

As a further step, the WLC and RMC models predicted that, as the IDL is shorter to some extent, the binding may be weakened rather than being strengthened, leading to an optimal linker length of about 43 residues, under which the binding affinity is the highest (Figure 3). It should be noted that these polymer models do not consider the protein volume and fluctuations of the end-to-end distance, which may cause some uncertainty that would be significant at a smaller linker length. It is also difficult to experimentally verify the situation at a short length, since the linker may not remain disordered. Anyway, the above results demonstrated that the entropic effect of the disordered linkers in VRN1 could effectively tune their binding affinity to DNA. By varying the linker length, it is possible to control the affinity and further influence the mesoscale phase separation process, as discussed below.

### 2.2. Simulations of Phase Separation under Different IDL Lengths

A previous study showed that multidomain proteins can function as stickers and spacers during the phase separation process [35]. The B3 domains in VRN1 resemble stickers to interact with DNA, while the IDL resembles spacers that contribute the effective local concentration. An experimental study revealed that the phase behavior of VRN1 and DNA transited from gel-like precipitates (*L* = 40) to liquid-like droplets (*L* = 100) and a clear solution (*L* = 160) when neutral linkers were used [44], where *L* refers to the number of linker residues. When a pair of positively and negatively charged patches were added to the linker, the phase behavior converged to the robust droplets, regardless of the linker length [44]. To better understand these phase transition processes, we performed coarse-grained Monte-Carlo simulations using LASSI [30,35,50,51], a lattice model developed by Pappu et al. (see Section 4). In this model, ρ and $\phi_{C}$ are two key quantities to be obtained from the simulations, which describe the density inhomogeneities and the extend of percolation, respectively. They can be used to distinguish between phase separation and gelation [35]. In this paper, we expect that if ρ exceeds 0.025, phase separation occurs; and if $\phi_{C}$ exceeds 0.5, gelation occurs. Each VRN1 variant protein was modeled as two beads, with an implicit linker connecting them. The reason to use the implicit linker is that the worm-like chain model or random-coil model can describe the IDL properties, by incorporating the excluded volume effect and nonspecific attraction into the persistent length [52]. This is also why the implicit linker models can be used to fit the experimental results in Figure 2. Every 10 base pairs (bp) of DNA were modeled as a bead. For DNA with 55 bp that was used in experiments, 5 consecutive beads were used. Every 10 residues in the linker were supposed to occupy 1 lattice unit length. Therefore, the linker length varied from 2 to 16 lattice unit length, with an interval of 2, corresponding to 8 simulation systems (*L* = 20, 40, 60, 80, 100, 120, 140, 160) to obtain a detailed picture of how phase behavior correlates with linker length. A total of 1000 protein and 250 DNA molecules were put into the simulation box to duplicate the experimental concentration ratio. Since electrostatic interactions play a key role, and the net charge of a single B3 bead and a DNA bead is +4 and −20, respectively, the contact energy between protein-protein, protein-DNA and DNA-DNA are set to be 0.4$k_{B}T_{0}$, $-0.4k_{B}T_{0}$ and $10k_{B}T_{0}$, respectively, where $k_{B}$ is the Boltzmann constant and $T_{0}=300$ K is the reference temperature. By varying the concentration, through modifying the size of the simulation box as well as temperature, the phase diagram of each system can be simulated.

**Figure 4:**
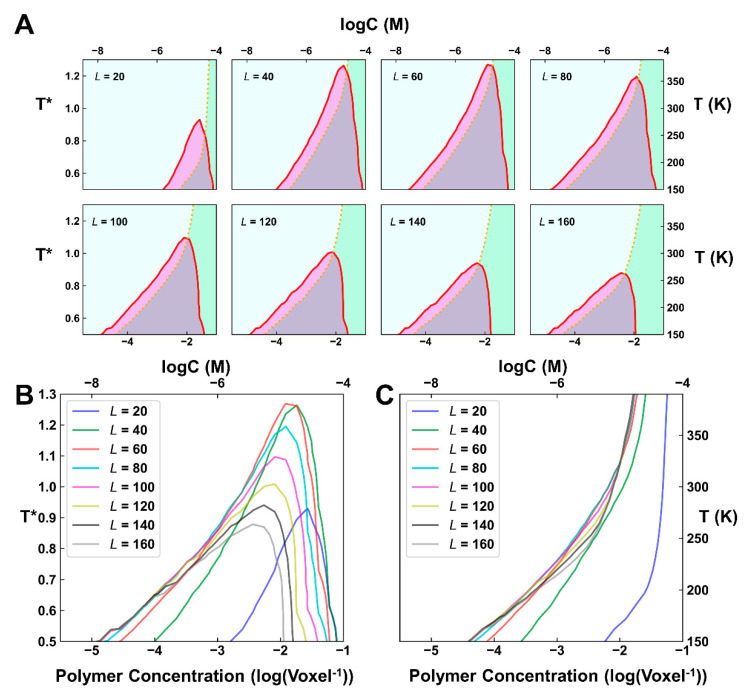
Influence of the neutral-linker length on the simulated phase behaviors. (**A**) Phase diagrams of the systems with different linker lengths (L=20~160). The red line shows the contour of ρ = 0.025 and the orange dotted line indicates the contour of $\phi_{C}$ = 0.5. The left cyan area indicates solution phase; the right green phase indicates gelation phase; the purple area indicates phase separation without gelation and the grey area indicates phase separation with gelation. $T^{*}=T/T_{0}$ refers to the reduced temperature. (**B**,**C**) Contour of ρ = 0.025 (**B**) and $\phi_{C}$ = 0.5 (**C**) for different systems. To make the interpretation more accessible, an approximation was made to transform a reduced concentration of $10^{-3}$ /Voxel to 1 μM.

The simulation results of the phase diagram under different linker lengths were summarized in Figure 4. It can be observed that as the linker length increased, the upper critical solution temperature (UCST) to undergo LLPS first increased and then decreased, and the concentration allowed to undergo phase separation globally became lower, indicating that the ability of LLPS first improved and later weakened. Gelation follows a similar tendency, as the concentration allowed to undergo gelation first decreased and then increased as the linker length grew, but the variation is far less significant. These simulations hint that if the linkers are short, two B3 domains prefer to bind to the same DNA in favor of forming network terminating dimers and oligomers, which weakens the ability of either gelation or phase separation, and if the linkers are too long, two B3 domains will become independent from one another and the enhanced local concentration effect becomes negligible, although gelation can still occur.

Evidently, the simulated phase separation behaviors correlated well with the binding affinity of VRN1-DNA. A higher affinity is more favorable for the occurrence of phase separation. Being judged from the UCST, the strongest phase separation occurs approximately between *L* = 40 and *L* = 60, which is close to the optimal linker length of *L* = 43, to achieve the highest affinity. A longer linker has a high entropic cost, which eventually inhibits phase separation. We also performed simulations with a different VRN1/DNA ratio, i.e., with 1000 protein and 500 DNA molecules. The obtained results showed a similar tendency (Figure S1). In short, these simulations indicated that linker length can effectively modulate the process of gelation, as well as phase separation. A shorter linker will prohibit the phase separation (and gelation), by forming network terminating dimers and oligomers, while a longer linker length will diminish the cooperativity of two adjacent B3 domains and disrupt phase separation. For an intermediate linker length, a strong tendency for phase separation may occur.

### 2.3. Simulations of Phase Separation for Systems with Charged Patches in Linker

The linker considered in the above simulations is neutral, so its effect is tuned by the length. On the other hand, naturally occurring VRN1 and VRN1-like proteins contain IDL with charge segregation. Adding charged patches on the linker can robustly maintain the liquid-like phase separation behavior of VRN1 variants and DNA [44]. As these patches made specific interactions with each other and with both the B3 domains and DNA, we next modeled the positively and negatively charged patches as two explicit beads, connected by the rest of the implicit linker. The contact energy among these components was listed in Table S1, according to the net charge of each bead type (the charged patch that was used in the experiments included +7 or −7 charged residues). Considering that each charged patch occupies one lattice unit length, the implicit linker length varied from 2 lattice units to 14 lattice units, with an interval of 4, which corresponds to 4 simulated systems (*L* = 40, 80, 120, 160).

**Figure 5:**
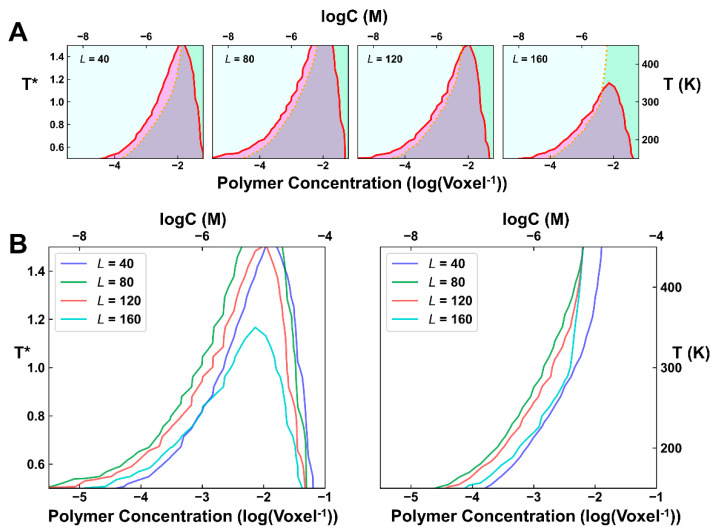
Phase behaviors for the systems with charged patches (a positively and a negatively charged patch) on the linker. (**A**) Phase diagrams of systems with different linker lengths. The used line and color scheme is the same as that in Figure 3. (**B**,**C**) Contour of ρ = 0.025 (**B**) and $\phi_{C}$ = 0.5 (**C**) for different systems.

The phase diagrams of these systems were simulated using the same settings as before. The obtained results showed that these systems had a much wider phase separation boundary and the UCST was much higher than the corresponding system with a neutral linker (Figure 5). Even for the system with the weakest LLPS ability (*L* = 160), the UCST was close to the neutral system with the strongest LLPS ability (*L* = 80), which was consistent with the experimental results [44] that charge segregation in the IDL ensures robust LLPS that does not depend on the IDL length. 

**Figure 6:**
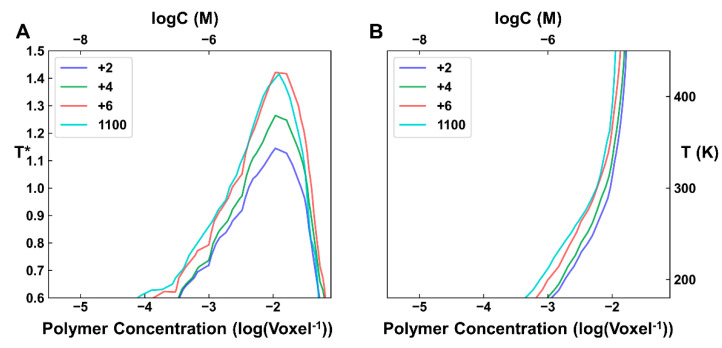
Influence of the linker patch charge neutral on the phase behaviors (*L* = 40). (**A**,**B**) Contour of ρ = 0.025 (**A**) and $\phi_{C}$ = 0.5 (**B**) for systems with a positively and a negatively charged patch. The label ‘+2’, ‘+4’, ‘+6’ refers to the absolute net charge for each charged patch. The label ‘1100’ refers to the linker architecture, in which two consecutive positively charged patches (that is ‘1’) connected to two consecutive negatively charged patches (that is ‘0’).

The naturally occurring charged patches in the linker often consist of several consecutive charged segments, while too short or too long charged patches are rare [44]. Therefore, we next investigated whether the too short or too long charged patches lead to weakened phase separation. To explore these situations, the linker with 40 residues was chosen as a model system and the absolute net charge for each charged patch was gradually decreased from +7 to +6, +4 and +2, to mimic the shortening effect (Table S1). We also constructed a ‘1100’ linker architecture in which ‘1′ refers to the original positively charged patches and ‘0′ refers to the original negatively charged patches, to mimic the elongation effect. The simulation results showed that the LLPS ability decreased, along with the decrease in the charged patch length (Figure 6). On the other hand, a system with longer charged patches can also slightly alleviate the LLPS ability, which can lead to a waste of resources in cells. This may be the reason why nature utilizes sequences with charge segregation patterns and modest lengths. 

## 3. Discussion

IDPs are widely spread in human cells and nearly half of human proteins are IDPs, possibly due to their many advantages, such as saving genome resources, achieving fast binding rates, and serving as flexible linkers to connect domains [53]. To perform complex functions, cells need to develop and evolve a method, possibly through phase separation, to precisely organize enormous molecules, on both spatial and temporal scales. Phase separation is a mesoscale event, involving multicomponent interactions to maintain the high-concentration condition. Although the underlying driving force can be electrostatic, hydrophobic, π-π, cation-π, dipole-dipole and non-specific interactions, they should be multivalent. A disordered linker is quite suitable for this demand. It can act as an entropic chain to modulate domain interaction or directly interact with the surroundings; both can induce LLPS. Recently, many transcriptional factors have been found to regulate gene expression through LLPS, in which the N- or C-terminal low complexity regions participate in the formation of large molecular machines and clusters of enhancers (the so-called super-enhancers) [54], with fast association and dissociation rates to accomplish high specificity with low affinity. Moreover, some pioneer transcriptional factors can modify the phase behavior or the accessibility of closed chromatins [55], possibly using the same strategy. It should be noticed that the IDL of VRN1 contains a considerable number of Y/F amino acids, as well as positively charged residues. It has been shown in our previous study [43] that the VRN1-DNA phase separation can be destroyed with a high concentration of salt, indicating that the electrostatic interactions play a dominant role here. The reason why the wild-type VRN1 protein contains a considerable number of Y/F amino acid residues and whether the cation-π interaction plays an important role in the VRN1-DNA LLPS should be analyzed in the future.

To reveal the underlying mechanism of IDL mediated biomolecular condensation, we applied a polymer chain model and phase diagram simulations to study the VRN1 system. We found that both the effective local concentration effect and charge segregation pattern could boost the ability of LLPS, which is consistent with experimental observations. This conclusion can be used to guide the design of linkers in constructing LLPS-enhanced functional systems, such as those containing functional modules, an example being fluorescent proteins or enzymes, which act as probes for translocation detection and chemoenzymatic microreactors [56]. Further research should explore such possibilities.

## 4. Materials and Methods

### 4.1. Fitting VRN1 Linker to WLC Model and RMC Model

The probability distribution function (${P(r}_{e})$) of the end-to-end distance ($r_{e}$) for the linker between two B3 domains of VRN1 was calculated using the WLC model and RMC model. For the WLC model, one domain can be expressed using the following equation [29,46,47,48,57,58]:

(2) $${P(r}_{e}{)=4\pi r}_{e}^{2}\frac{1}{N_{A}}{\left(\frac{3}{{4\pi l}_{p}l_{c}}\right)}^{\frac{3}{2}}\exp\left(-\frac{{3r}_{e}^{2}}{{4l}_{p}l_{c}}\right)\left(1-\frac{{5l}_{p}}{{4l}_{c}}+\frac{{2r}_{e}^{2}}{l_{c}^{2}}-\frac{{33r}_{e}^{4}}{{80l}_{p}l_{c}^{3}}-\frac{{79l}_{p}^{2}}{{160l}_{c}^{2}}-\frac{{329r}_{e}^{2}l_{p}}{{120l}_{c}^{3}}+\frac{{6799r}_{e}^{4}}{{1600l}_{c}^{4}}-\frac{{3441r}_{e}^{6}}{{2800l}_{p}l_{c}^{5}}+\frac{{1089r}_{e}^{8}}{{12800l}_{p}^{2}l_{c}^{6}}\right)$$

where $l_{p}$ represents the persistent length of the linker, which is set to 3.0 Å, and $l_{c}$ is the total contour length of the linker, which is the multiplication of the residue number in the linker and the average distance between the adjacent $C_{\alpha }$ (3.8 Å). $N_{A}$ is the Avogadro constant. For the RMC model, one domain can be expressed using the following equation [28,49,59]:

(3) $${P(r}_{e}{)=4\pi r}_{e}^{2}{\left(\frac{3}{2\pi }\right)}^{3/2}\frac{1}{N_{A}{2(\langle r^{2}\rangle ){}^{1/2})}^{3}}\exp(\frac{{-3r}_{e}^{2}}{2(\langle r^{2}\rangle ){}^{1/2}{)}^{2}})$$

where $\langle r^{2}\rangle {}^{1/2}$ is the root mean square of $r_{e}$, which equals to $\sqrt{{2l}_{p}l_{c}}$. The effective local concentration for the formation of a DNA-protein complex is proportional to ${P(r}_{e})$, given by the following equation:

(4) $$C_{\mathrm{eff}}{=p}_{0}\frac{{P(r}_{e})}{{4\pi r}_{e}^{2}}$$

where $r_{e}$ is assigned to the distance value between the B3 domain ends in the VRN1-DNA complex to be connected by a disordered linker (see Figure 1) and $p_{0}$ is a proportional factor. It is noted that $r_{e}$ in Equation (4) is independent of the linker length and the VRN1-DNA binding affinity. The linker length affects $C_{\mathrm{eff}}$ merely via $l_{c}$ in Equations (2) and (3). $C_{\mathrm{eff}}$ plays an essential role in the linker’s effect, since it further relates the VRN1-DNA binding affinity to the affinity between a single B3 domain and DNA, as shown in Equation (1).

### 4.2. Lattice-Based Coarse-Grained Monte-Carlo Simulations of Phase Diagrams

Phase diagram simulations were performed using LASSI, a lattice simulation engine for sticker and spacer interactions, developed by Pappu et al. [30,35,50,51]. LASSI performs Monte-Carlo simulations using a simple lattice model. The molecular evolution is driven by a variety of designed Monte-Carlo moves, including chain pivot, translation, rotation moves of both the individual molecules and clusters of molecules and so on. In our study, each VRN1 protein was modeled as two beads, with an implicit linker connecting them by distance restraint. Every 10 base pairs (bp) of DNA were modeled as a bead. For DNA with 50 bp in simulation, 5 neighbor beads were used. Every 10 residues in a linker were supposed to have 1 lattice unit length. The concentrations (which were titrated by changing the simulated box) and temperature were set as the independent variables. The contact radius used followed the default definition in LASSI, which suggests that the beads are considered to be adjacent to one another if they are within a lattice distance of $\sqrt{3}$. The overlap potential was the same as the contact energy. For each system, two independent simulations were conducted, each of which consisted of $10^{9}$ MC steps, after ${5\;\times \;10}^{6}$ equilibration steps. To further elucidate the influence of the charged patches on phase separation, the positively and negatively charged patches inside the linker were modeled as an explicit bead, connected by the rest of the implicit linker. 

## Figures and Tables

**Table 1:** The fitting results of WLC and RMC with experimental data.

Name	Apparent *K*_d_ from Ref. [44] (nM)	*C*_eff_ (mM) ^a^	RMC-*K*_d_	RMC-*C*_eff_	WLC-*K*_d_	WLC-*C*_eff_
Single B3	753					
PSN40	20	0.0284	21.4	0.0265	21.4	0.0265
PSN60	25	0.0227	21.8	0.0260	21.7	0.0261
PSN80	26	0.0218	24.9	0.0227	24.9	0.0227
PSN100	30	0.0189	29.2	0.0194	29.2	0.0194
PSN120	32	0.0177	34.1	0.0166	34.1	0.0166
PSN160	52	0.0109	45.2	0.0125	45.2	0.0126

^a^: the $C_{\mathrm{eff}}$ was calculated using Equation (1).

## Data Availability

The LASSI simulation files for reproducibility are uploaded in LASSI_files.zip.

## Supplementary Materials

The following supporting information can be downloaded at: https://www.mdpi.com/article/10.3390/ijms23094594/s1.
