# Supplementary material for: The Regulatory Roles of Intrinsically Disordered Linker in VRN1-DNA Phase Separation
Source: Int J Mol Sci. 2022 Apr 21;23(9):4594. doi: 10.3390/ijms23094594 (PMC9106000; doi:10.3390/ijms23094594)
Supplement: Supplementary file 1 [file ijms-23-04594-s001.zip › ijms-1665755-supplementary/manuscript-SI-220416.pdf]

The Regulatory Roles of Intrinsically Disordered Linker in VRN1-DNA Phase Separation

Qiaojing Huang [1], Yanyan Wang [2], Zhirong Liu [1,*] and Luhua Lai [1,2,3,4*]

[1] Beijing National Laboratory for Molecular Sciences (BNLMS), State Key Laboratory for Structural Chemistry of Unstable and Stable Species, College of Chemistry and Molecular Engineering, Peking University, Beijing 100871, China; chemhqj@pku.edu.cn (Q.H.)

[2] Peking-Tsinghua Center for Life Sciences, Peking University, Beijing, 100871, China

[3] Center for Quantitative Biology, Peking University, Beijing 100871, China

[4] Research Unit of Drug Design Method, Chinese Academy of Medical Sciences (2021RU014), Beijing 100871, China

* Correspondence: LiuZhiRong@pku.edu.cn (Z.L.); lhlai@pku.edu.cn (L.L.)

Supplementary material

Figure S1: Contour of ρ = 0.025 and ϕ_C = 0.5 for different neutral linker length systems with the concentration ratio of protein/DNA = 2/1.

Table S1: The contact energy among different components.

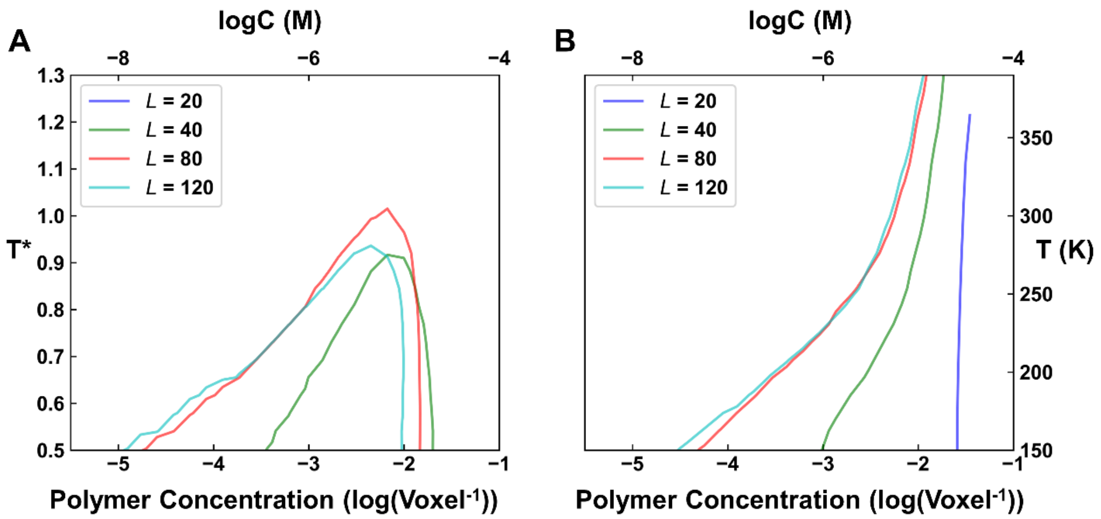

Figure S1. Contour of (A) $\rho = 0.025$ and (B) $\phi_C = 0.5$ for different neutral linker length systems with the number of protein : DNA = 1000 : 500.

Table S1. The contact energy among different components[a]. (unit: $k_B T_0$)

	protein bead	DNA bead	positively charged patch	negatively charged patch	+2 charged patch	+4 charged patch	+6 charged patch
protein bead	0.4	-2.0	0.7	-0.7	0.2	0.4	0.6
DNA bead	-2.0	10.0	-3.5	3.5	-1.0	-2.0	-3.0
positively charged patch[b]	0.7	-3.5	1.2	-1.2	-	-	-
negatively charged patch	-0.7	3.5	-1.2	1.2	-	-	-
+2 charged patch[c]	0.2	-1.0	-	-	0.1	-	-
+4 charged patch	0.4	-2.0	-	-	-	0.4	-
+6 charged patch	0.6	-3.0	-	-	-	-	0.9

[a]: The contact energy here is mainly determined by the electrostatic interaction between different components. The net charge of a single B3 domain in VRN1 is +4, and the net charge of a single DNA bead is -20. So the ratio of contact energy among protein bead - protein bead, protein bead – DNA bead, and DNA bead – DNA bead, is approximately 16 : -80 : 400, which is reduced to 0.4 : -2.0 : 10.0.

[b]: The charged patch that was used in experiments includes +7 or -7 charged residues, and the net charge of a positively or negatively charged patch is +7 or -7, respectively. The ratio among protein bead, DNA bead, positively charged patch, and negatively charged patch is determined as above.

[c]: The artificially constructed +2. +4, or +6 charged patch is supposed to contain +2, +4, or +6 charged residues in a single charged patch, which combines with the -2, -4 or -6 charged patch to form three systems with decreased charged residues in a single charged patch. The ratio among protein bead, DNA bead, positively charged patch, and negatively charged patch in these system is also determined as above.
